# Health promotion focusing on migrant women through a community based participatory research approach

**DOI:** 10.1186/s12905-021-01506-y

**Published:** 2021-10-16

**Authors:** Cecilia Lindsjö, Katarina Sjögren Forss, Christine Kumlien, Margareta Rämgård

**Affiliations:** Department of Care Science, Faculty of Health and Society, Malmö University, Jan Waldenströms Gata 25, 20506 Malmö, Sweden

**Keywords:** Story-dialogue method, Health promoter, Empowerment, Health literacy, Social determinants of health

## Abstract

**Background:**

Migrants are often more vulnerable to health issues compared to host populations, and particularly the women. Therefore, migrant women’s health is important in promoting health equity in society. Participation and empowerment are central concepts in health promotion and in community-based participatory research aimed at enhancing health. The aim of this study was to identify conditions for health promotion together with women migrants through a community-based participatory research approach.

**Methods:**

A community-based participatory research approach was applied in the programme Collaborative Innovations for Health Promotion in a socially disadvantaged area in Malmö, Sweden, where this study was conducted. Residents in the area were invited to participate in the research process on health promotion. Health promoters were recruited to the programme to encourage participation and a group of 21 migrant women participating in the programme were included in this study. A qualitative method was used for the data collection, namely, the story-dialogue method, where a process involving issue, reflections and actions guided the dialogues. The material was partly analysed together with the women, inspired by the second-level synthesis.

**Results:**

Two main health issues, mental health and long-term pain, were reflected upon during the dialogues, and two main themes were elaborated in the process of analysis: Prioritising spare time to promote mental health and Collaboration to address healthcare dissatisfaction related to long-term pain. The women shared that they wanted to learn more about the healthcare system, and how to complain about it, and they also saw the togetherness as a strategy along the way. A decision was made to start a health circle in the community to continue collaboration on health promotion.

**Conclusions:**

The community-based participatory research approach and the story dialogues constituted an essential foundation for the empowerment process. The health circle provides a forum for further work on conditions for health promotion, as a tool to support migrant women’s health.

## Background

Migrants are at higher risk, compared to populations of host countries, of acquiring diseases [1]. Health vulnerability is thus greater among migrants, particularly among women, and may consequently entail specific healthcare needs [2]. Mental issues, such as anxiety and depression, have been shown to be more common in a migrant population compared to the host population [3]. Furthermore, research among Syrian migrants living in Sweden revealed that mental health problems were more common in Syrian women than in their male counterparts [4]. Similarly, another study on self-rated health among Iraqi migrants in Sweden, showed that Iraqi migrants, and in particular Iraqi women, tended to rate their health poorer than the native Swedish population [5]. In addition, women from Iraq had a lower belief in their ability to promote their own health compared to Swedish women and engaged less in physical activity [5]. Health interventions intended to help the general patient population, such as physical activity on prescription, may not be compatible with the situation of migrant women [6]. In summary, the health situation of migrant women can be compared neither with that of migrant men nor with that of women of host countries. Therefore, according to previous research, one could argue that migrant women’s health is important to consider when promoting health equity in society.

Health inequity is linked to social determinants of health, which should be kept in focus when working for health equity [7]. The Swedish Commission for Equity in Health and the Malmö Commission have emphasised the importance of participation and empowerment in enhancing health [8, 9]. Thus, the reports of the commissions can be said to be in line with the central concepts of health promotion, i.e., empowerment, participation, holism, cross-sectoral collaboration, equality, sustainability and multi-strategy [10, 11].

According to the World Health Organization (WHO), health promotion is ‘*the process of enabling people to increase control over, and to improve, their health*’ [12]. It includes a broad range of measures regarding how to influence individuals’ health behaviour as well as focusing on social structures [10, 11]. Thus, health changes can be conducted on different levels, individual, community or public, as well as on several levels simultaneously, often with even more impact [13]. Moreover, the interplay between levels is important, connecting individuals to their context [11]. Worldwide, no commonly accepted theory of health promotion exists, despite numerous theoretical frameworks, but the central concepts referred to above are a foundation [10]. Previous studies focusing on health promotion among migrant women have shown positive results. For example, a study on the cultural adaptation of a health-promotive intervention showed perceived improved health [14], and another study, on a salutogenic health-promotion programme, showed trends pointing to better self-esteem and reduced stress [15].

The central concepts of health promotion are fundamental in the programme Collaborative Innovations for Health Promotion, wherein this study is situated, and the approach of Community-Based Participatory Research (CBPR) was applied in the programme [16]. Wallerstein et al*.* have defined CBPR as: ‘collaborative efforts among community, academic, and other stakeholders who gather and use research and data to build on the strengths and priorities of the community for multilevel strategies to improve health and social equity’ [17]. In a CBPR project the community members are part of the research process as partners compared to community-placed research where the researchers conduct the research in a community without involving community member in the research [17]. One of the primary objectives of CBPR programs is to reduce health inequity through community empowerment. Favourable conditions for participation will substantially affect the outcomes of projects aiming to reduce health inequity through community empowerment [17]. CBPR is about addressing structural inequalities and social injustice, together with the community members who are struggling with these issues [18]. Through social changes, inequalities in health can be reduced [19]. Engagement in the community is therefore essential to achieve empowerment and take action [18]. CBPR is one way of doing participatory action research and the approach has been developed from and inspired by the participatory action research paradigm [19, 20], where a cyclic process is applied, moving from problem to reflection to action [21]. The participatory research approach has commonly been used in research regarding marginalised groups as a defiance to oppression and a questioning of power relations of knowledge [20]. In the process of democratisation and the act of change, Paulo Freire, among others, emphasises the importance of, and has developed the teaching of, critical thinking, which initiates the empowerment process [20, 22]. To enable critical thinking and empowerment, participation is essential [22]. The CBPR approach can bring together research, theory and practice and is suitable for marginalised populations lacking involvement in the community [23]. The CBPR approach is often used in the context of health inequalities when a subpopulation is differently affected by health conditions and poorly understood compared to the general population [24].

The programme within which this study was included takes as its point of departure the community members’ own perception of their needs of health promotion, initiating a focus on migrant women’s health. CBPR for community health promotion has been previously used in health interventions focusing on physical activity and nutrition to prevent chronic diseases, among migrant women in the USA [25]. Congolese migrant women in the USA have also, together with researchers, used the CBPR approach for health promotion, resulting in photo and story sharing on themes such as healthcare system issues, social support, and daily experiences of health [26]. Furthermore, review studies have concluded that community work for migrant women can include the initiation of dialogue and collaboration to increase community social support, which can prevent isolation as well as improving mental health and access to healthcare [27]. Many stressors, such as housing or work, may affect health among migrant women, and social support may work as a resilience strategy in handling various situations [28].

Promoting health among women is important from a societal perspective in order to reduce health inequality [29]. Moreover, women should be involved in the construction of the knowledge needed to promote their health, especially women with different needs than the majority [29]. Migration is a global phenomenon, and, while acknowledging previous research, studies using the CBPR approach are needed to understand the conditions for health promotion in a North European context. The above-mentioned studies with a CBPR approach have been conducted in the USA [25, 26], a context that might differ from the North European one. For example, in the USA, women migrants were concerned about access to health insurance and consequently access to healthcare, whereas in Sweden migrants are entitled to healthcare when they get a residence permit and an address in Sweden [26, 30]. Therefore, the aim of this study was to identify conditions for health promotion together with women migrants in a Swedish context through a CBPR approach.

## Methods

### Design and setting

This study was part of the programme Collaborative Innovations for Health Promotion. The CBPR approach was applied, which was suitable since the programme was conducted in a socially deprived area of Malmö, Sweden, where parts of the population may be marginalised. This area is classified as a socially vulnerable area. According to the Swedish police authority, a socially vulnerable area is defined as an area with a population with low socioeconomic status and widespread criminality that affects the local society [31].

Initiatives for researching issues regarding health equity in this area were taken by researchers at Malmö University, and a further description of those initiatives is presented elsewhere [16]. In brief, residents were invited to so-called Future workshops in 2016–2017 and together with researchers and other actors they developed an action plan, recognising obstacles to the promotion of residents’ health. Using a multisectoral approach, solutions for health promotion activities were elaborated [16]. A co-creative lab focusing on women’s health, and included in the programme, was initiated by women migrants, primarily from countries of the Middle East, though the group was open to women with any ethnic origin. Activities mainly focusing on social inclusion, started for women within the community.

To facilitate participation, lay health promoters from the community were recruited into the programme. Their role implies (a) a similar migration background as the community being served, (b) knowledge and understanding of the society and the systems of health and social services in the new country as well as in the one they have migrated from, and (c) the ability to use that knowledge and understanding to help the community participants [32]. Community health workers have previously been researched as an option for engaging in health promotion among women migrants, and their role also enables addressing social determinants of health [32]. In this study, the role of the health promoter was to initiate actions with regard to health-promoting issues according to the action plan. The health promoter could translate language-wise but also culturally, explaining behaviours to both researchers and participants. Moreover, the health promoter kept up the contact between participants and researchers, reached out to and recruited new participants, and informed about activities. The health promoters contact with participants, both in groups and individually, was kept up by using social network applications, short message service (SMS) and phone calls. To have more personal contact besides general messages is inevitably demanding for the health promoter, while creating a safe atmosphere for the participants.

Issues that had been identified in the Future workshops had led to actions with and for the women in the co-creative labs. In the spring of 2018, regular meetings were held during a trust-building phase until October 2018 and after this a new cycle in the research process was to begin.

### Study participants and data collection

Data was collected using the story-dialogue method. The story-dialogue method, which was developed by Labonte, Feather and Hills, is a technique that can be used to explore and acknowledge a person’s stories and experiences, in a structured way that makes them useful for health promotion [33]. Reflection is in focus in the method with the purpose to reach a shared understanding between participants and practitioner/researcher [33]. The story-dialogue method has previously been used among migrants [34]. The use of stories for knowledge development has been emphasised as suitable for reaching poor communities where traditional research methods can be suboptimal, for example, within populations where little research is compiled and researchers do not know which questions to ask, or when aiming for consciousness-raising among women [33]. Using participants’ own stories can help them find their own voice, thus forming the basis for opposing social structures [35, 36], and sharing one’s own story is part of the empowerment process [33]. Stories engage emotions which can impact the motivation for action [20]. In addition, the structure of the story-dialogue method offers the opportunity to move from the issue to actions, through reflection, together with participants [33], which is consistent with the action research process [21]. The exchange of perspectives is essential to develop each person, since one person’s understanding does not fully overlap with other persons’ understanding and is formed according to experiences [20]. It is therefore essential to create a forum for dialogue within participatory practice [37].

The study took place in social meeting places in the community that the women were familiar with. To create a comfortable atmosphere the researchers visited the women´s area and the place where the study was conducted on several occasions before starting the research to build trust. Women from the community health program was invited to take part of the research and could communicate in their own language. Local health promotors distributed written invitations to women in the co-creative lab. The women were then invited to meet the researchers to receive oral information, translated by the health promoters, about the research study and had opportunity to ask questions about the research. The study population from the co-creative lab can be seen as a natural group that can be useful to maximise interaction between participants in reflection [38], also due to the heterogeneity in the group since women from different middle eastern countries differ in culture.

The women who wanted to participate were divided into three groups and the dialogues took place in October 2018. Thus, three story dialogues were conducted with 5–8 women in each group, in total 21 women. Two of the women wanted to stay anonymous and did not want to share details about themselves. The women originated from countries of the Middle East, such as Iraq, Lebanon, Palestine, Yemen, Syria and Jordan, and from African countries, such as Morocco and Sudan. They had been living in Sweden between 1 and 34 years (median 15 years) and the age of the women was between 26 and 75 years (median 46 years). There was a wide variation in the source of income, including income from work, study grant, pension or social security benefits. The educational background also varied, from 5 to 16 years of education (median 9 years).

The dialogues took place in a closed room in a community space, familiar to the participants. The health promoter assisted in translating from Arabic into Swedish and vice versa, since the researchers did not speak Arabic and many of the participants spoke limited Swedish. All dialogues were audio recorded and two or three of the authors were present during the dialogues, one observing and taking notes and one or two facilitating the dialogue.

Before each dialogue, one participant in each group prepared a story on the topic of a health issue encountered in daily life. The method follows a circular structure to deepen a story that is told. The first part consists of listening to a story, and this is followed by a reflection circle and a structured dialogue. The reflection circle gives the participants opportunity to reflect and share with the group one by one how the story that has been told connects to themselves [33]. The structured dialogue includes questions encouraging the participants to further describe, explain and, finally, synthesise and come up with possible actions [33]. This is followed by a review of notes taken during the dialogue, and it ends with the creation of insight cards, that is, statements based on the significant notes from the dialogue [33]. The process of the story-dialogue method is expected to lead to actions which can later be evaluated [33].

The method was modified to be suitable to and accepted by the study population. Others who have previously used the method have also modified it according to their needs [39]. For example, in this study, due to the participants’ limited time, each dialogue included only one story. Additionally, because of language barriers, the insight cards and themes were written by the researchers when none of the participants volunteered.

### Analysis

Second-level synthesis was used as inspiration for the analysis [33]. This method consists of moving from individual stories of experiences to a more general material, thus developing the knowledge [33]. The analysis was conducted together with the participants and primary themes and sub-themes were identified. Then, all data relevant to the purpose of the study were extracted and transcribed by the researchers, and the material was subsequently analysed to find new themes and sub-themes that had not been identified by the participants. The analysis was structured according to the phases of action research, look-reflect-act [21], which fits well with the process of the story-dialogue method [33]. The ‘look’ phase includes the description, the reflection phase includes explaining and synthesising and, finally, the act phase includes the actions identified in the dialogues. Themes identified by the participants framed the analysis of all the material, and some themes from the three story dialogues overlapped. NVivo qualitative data analysis software (version 12) was used as a tool to keep the texts and codes in order [40].

## Results

Two main health issues, mental health, and long-term pain were reflected upon during the dialogues, and the process of action research, including the phases look, reflect and act, was followed (Table 1). Based on this, two main themes were identified in the process of analysis: Prioritising spare time to promote mental health and Collaboration to address healthcare dissatisfaction related to long-term pain.

**Table 1:** Themes and sub-themes structured according to the process of the story-dialogue method

Prioritising spare time to promote mental health		Collaboration to address healthcare dissatisfaction related to long-term pain
Mental health issues were considered to be a trajectory to other health problems	Look	Challenges of long-term pain entails other health issues
Migration and household work were thought to be associated to mental health issues	Reflect	Household work as the root to pain and dissatisfaction with the healthcare
Moving forward through prioritising self and teaching children about gender equality	Act	Working together to make a change, if not for oneself then for someone else

### Prioritising spare time to promote mental health

The sub-themes within this theme were labelled as follows: Mental health issues were considered to be a trajectory to other health problems, Migration and household work were thought to be associated to mental health issues, and Moving forward through prioritising self and teaching children about gender equality (Table 1).

#### Mental health issues were considered to be a trajectory to other health problems

In the ‘look’ phase, the women defined mental health issues. Their stories of mental health issues were frequently about stress or depression, phenomena associated with their daily lives and struggles to adapt to a new country and to the unstable situation of migration. Several women found that they had similar experiences and talking about this made them feel sad. The women also associated mental health issues to both weight loss and weight gain which led to other, physical, health issues, such as pain, breathing problems, sleep deprivation and decreased ability to move.

Stress affects the whole body. Mental health affects physical health. It causes pain. (Story dialogue 3)

#### Migration and household work were thought to be associated to mental health

The main reasons for the women’s mental health issues were identified, thus constituting the phase of reflections. When the women reflected with each other in the group, they considered the reasons for their health issues to be outside of their control.

The first reason the women reflected upon was the situation of migration. Both physical and mental symptoms experienced during the process of transfer from one country to another and after arrival in the new country, were described. The physical symptoms could be breathing problems, tension, pain and headache, but were thought to be related to the depression. The women also reflected on specific issues in the migration situation which they thought influenced their mental health. One woman talked about the change in climate when she had moved from a warmer country to a cold one. Another woman described how the cold made her feel tired and depressed. Furthermore, unstable housing and the change of housing when migrating were highlighted.

We used to live in big houses with a bottom floor [quadrangles], but here we must live in apartments in high-rise buildings. (Story dialogue 3)

The uncertain migration status, that is, not knowing whether they would get permission to stay or not, brought about a stressful situation that led to pain and mental health issues. It was highlighted that the society from where they came was completely different from the Swedish society, and fear of the new culture as well as difficulty to integrate were issues encountered. One woman argued that the relationship with their husbands was one reason for women not being able to integrate in the new society. The men had the power to forbid the woman to meet with other people or go out of the home, and this had an impact on their mental health. But some others in the group argued that not all men were alike and that it takes some time to familiarise yourself with the equality in a new system.

I think that my husband has influenced my life negatively. He wants to live here in Sweden, and he knows it’s a completely different culture, but he doesn’t try to balance between the two cultures. I think it’s normal when you move to another society that you try to adjust to the new society. (Story dialogue 3)

The other reason for mental health issues was associated to the responsibility women have in the household, with little support from the husbands. One woman said that before the marriage the women hope to share responsibility for household duties with their husband. However, husbands sometimes work away from the home many hours per day, with little possibility to accomplish household work. This is necessary due to the family’s economic situation, leaving those husbands that want to help with household duties with no option. Additionally, the women argued that as migrant women they are alone here in the new country with no support from relatives. Therefore, the women must handle the children and the household, as well as completing their own education.

I think that women have a lot of responsibility at home and they need to take care of their children. /… / And a lot of women actually suffered depression after giving birth. (Story dialogue 1)

The role of a woman was described as being a leader of the family and hence they felt that they needed to be strong, so as not to rock the boat. The many commitments led to an untenable position, and not completing the household work perfectly was associated with guilt. With the responsibility for the household resting solely on them, the women had little energy remaining for themselves and did not prioritise themselves.

In most cases women think mostly of their children, to take care of them in a correct way. It takes a lot of energy from the woman and then sometimes she forgets herself to give them [the children] a lot of time. (Story dialogue 1)

#### Moving forward through prioritising self and teaching children about gender equality

After the reflections, a phase with thoughts of actions followed. A successful change described by one woman was given credit by other women and inspired them. One of the women described how she had changed her situation of ill health. She had found motivation in considering the risk of secondary health issues, and in previous experiences of health issues in the family.

When I hear her story, I want to do the same because I also have family and children. I also have [over]weight. Therefore, when I listen to her, I think a lot about myself because I want to get better for myself too. (Story dialogue 1)

They reasoned that to take care of their children they first had to help themselves to health. To bring about change, accountability to oneself and a change of mindset were necessary. Additionally, to maintain changes, the support of a belief, for example, in God, was important.

The women reflected on actions aimed at teaching their children about gender equality to accomplish change for the next generation. They wanted both to make their daughters strong and to equip their sons for the future to take care of their own home. Therefore, it was important not to make a difference between sons and daughters when assigning household duties.

Must teach them about gender equality. /…/ Must treat the two of them equally at home. (Story dialogue 1)

However, this could be difficult, since some sons refused to help in the household, thinking it was the women’s duties, the women argued. To adapt to a behaviour of shared responsibility, maybe smaller children could be asked to help, but older children needed male role models, they said. But one woman had told her sons that the division of duties was an old thinking. Furthermore, it was mentioned that it was important to find a balance between work away from home and household work.

To benefit themselves, the women decided to try to find time for themselves to cope with their stressful situation, time that could be used to engage in physical activities.

I want to have special time for myself. For example, one hour a day or two hours a day to start gymnastics, to do something. (Story dialogue 1)

A women’s group was suggested by the researchers and adopted by the women. To make time for such a group, the women reasoned, they had a responsibility to themselves to let go, for example, by allowing the fathers to take responsibility. Some were eager to try this idea, while others did not have any chance to do so.

Additionally, the women wanted to meet with a psychologist to advise them on how to act. It was also mentioned that maybe the men need a supporting group when arriving in a new country, such groups being normally for women.

### Collaboration to address health care dissatisfaction related to long-term pain

The sub-themes within this theme were labelled as follows: Challenges of long-term pain entails other health issues, Household work as the root to pain and dissatisfaction with the healthcare, and Working together in order to make a change, if not for oneself then for someone else (Table 1).

#### Challenges of long-term pain entails other health issues

Within the ‘look’ phase, long-term pain was identified as a health problem the women encountered in daily life. Many participants could relate to the situation since they had experienced similar pain or had close relatives who had. Long-term pain often led to other problems, such as sleep deprivation and decreased ability to move, as well as being psychologically challenging and sometimes leading to depression. The pain did not just affect the women but also their families, though pain was concealed in front of the children to calm them.

I don’t want to show my children that I’m sick, or weak. I don’t want to show them because they are sensitive. Therefore, I always say to them that I’m very well. Because that makes them a bit calmer as well. (Story dialogue 2)

#### Household work as the root to pain and dissatisfaction with the healthcare

In this phase, the women reflected on reasons for pain, and reflections on the healthcare were shared. The women talked about the effect household work had on the pain they perceived. Many Arabic women have pain and the reason for this, they said, was that Arabic women are responsible for the household. One woman reflected that women do not work outside the home, only in the household with cleaning and cooking. They do not engage in any physical activity and have not done so while they were young, and now with age this shows.

I know her (referred to the storyteller), pain. All Arabic women [suffer from] pain. Why? Not working, no gym, only working in the home. (Story dialogue 2)

One participant had heard from a physiotherapist that she was weak because she did not exercise. The importance of physical activity and food was highlighted by the women in relation to pain and decreased ability to move. Memories of relatives’ situations had made them realise how important physical activity is.

Self-determination was thought by the women to help them out of the situations they were in. One woman explained that she had done everything she could to prevent herself from ending up in a wheelchair and to avoid operation. Another woman said that it was important for her to continue to teach to prevent depression. She could not work as before, due to her long-term pain, but instead worked on a voluntary basis, and in this way, she helped herself. It was said that one must help oneself and not wait for the doctor to help.

Okay, I like to continue to work as an Arabic teacher. That’s what I want, and I feel happy and proud to work. /…/ I continue to work on a voluntary basis. To feel good and not get depressed because of not being able to handle my life and my job. I need to think how I will help myself. (Story dialogue 3)

Thus, they described how they followed recommendations from the healthcare to help themselves. Weight loss, new shoes, physiotherapy, water aerobics and pain relief treatment were some of the recommendations they had followed, and some had helped. One of the women said she got good help from the doctor she had met and wanted to continue with a follow-up since she was not fully recovered, but, disappointingly, the doctor refused.

Moreover, the women reflected upon difficulties they had encountered within the healthcare in relation to the pain they perceived. Many had the same problem and thought that they had not got proper help from the healthcare. This, one of the women explained, led to a feeling of hopelessness. Another woman said that it brought a feeling of being imprisoned and others went on to say that it brought about mental health issues and physical consequences. They identified some of the difficulties they had encountered. Some of the women highlighted diagnostic difficulties, which were said to lead to a poor medical treatment of their issues. Long waiting times, together with ambiguity regarding the responsibility for one’s care, led to a dissatisfied and uncertain perception of the healthcare. The ambiguity regarding responsibility was thought to be due to both high staff turnover and the fact that physicians did not take into account decisions made by another physician. Too few and inexperienced physicians, especially specialised physicians, were seen as reasons for the difficulties within the healthcare. The women also reasoned that the system was to blame for the difficulties, not the single physicians, as they were required to follow the rules and think economically according to their organisation. Access to Arabic-speaking physicians had shown that the language barrier could not entirely explain the difficulties the women perceived within the healthcare. Instead, the bureaucracy made the women feel that the physicians did not care genuinely about them as patients.

I’ve had enough, they don’t work from the heart, they work to get paid. A physician doesn’t work because he’s a physician and wants to help. (Story dialogue 2)

The difficulties the women encountered within the healthcare, in association with their long-term pain, made them compare the Swedish healthcare to the healthcare in their home countries. This caused them to question and wonder at the long waiting times in emergency care or for results of a simple blood test. However, they also said that in their home countries only those with money were entitled to healthcare; the rest died.

#### Working together in order to make a change, if not for oneself then for someone else

In the ‘act’ phase, the dialogues stressed the importance of acting within the healthcare system. Many suggestions about what should be done were put forward: the doctors need more experience, access to specialists should be easier, healthcare centres need to be more serious about helping their patients, the government needs to invest more in healthcare centres, and more time is needed to meet with doctors. In relation to this, the women arrived at the conclusion that they themselves needed to understand the system better. It was mentioned that politicians should be addressed, but it was also said that members of the community could help each other.

The women wanted to protest about the healthcare but did not have the resources to do so. To accomplish change, they decided to write something together. Different opinions about this were shared, involving a feeling of hopelessness and a lack of trust that it would lead to a change, but also a feeling that maybe things can get better for someone else.

If one thinks that if one had complained, it might get better for other people. Sometimes one must think like that, why not complain to make them improve. So that what happened to me will not happen to someone else. (Story dialogue 2)

They decided to accept an initiative from the researchers regarding a health circle, to continue to work with the ideas that had been brought up during the dialogues. The attitudes of the participants differed, in that some of them wondered if the health circle would be helpful, while others welcomed the idea.

## Discussion

The findings identified several important conditions for health promotion among migrant women. Through their stories the women recognised health issues of concern. In the dialogues, the women reflected on reasons for and consequences of those health issues. The stories and reflections were also closely linked to social determinants of health, such as gender roles, housing and the accessibility of the healthcare system. Suggestions on actions emerged from the reflections. Thus, the CBPR approach and the story dialogues offered the women a forum for reflection and a common plan to continue the process of health promotion.

The women in our study reasoned that they must rely on themselves to change their own health. The women felt let down by the healthcare; hence, a need to understand the healthcare system better was emphasised. In previous literature, migrants also acknowledge the need of more information about the healthcare system in host countries [41, 42]. Moreover, health literacy has been shown to be low among migrants, that is, they often have less knowledge about health information, how to access it and how to apply it [43]. Additionally, the social gradient influences health literacy, in that it is lower in groups with lower education and social status and less financial resources, and therefore the social gradient is important for health-promotive work [44]. Thus, it is not merely the migrant status that decides the level of health literacy, which implies that migrants need to be treated as a heterogenous group [43]. Having said that, in this study, the median years of education among the women were 9 years, but ranging from 5 to 16 years, and it was common to depend on social security, but other sources of income also occurred. However, some consensus was apparent in the group regarding not having resources to complain about the healthcare. Consequently, migrant women’s voices may not be heard in the healthcare system, thus not contributing to policy changes. Based on our work, one could argue that in a population of migrant women, changes in health literacy are closely connected to the social determinants of health. Policy changes in healthcare are needed, as well as a more culture-sensitive behaviour from professionals in healthcare. Policy changes can be related to a higher extent of health literacy, which is also connected to the ability to change social determinants of health [45]. In the acting phase of this study, the suggestion of a health circle, where health issues could be discussed and health-related information brought forward, was adopted by the women. Health literacy may increase among migrants by integrating recommendations such as adapting information, assuming that health literacy may vary, and distributing information relevant to the group [42]. Following such recommendations, a dialogue with the people concerned would be appropriate, as in this study, where the women were invited to frame health promotion for themselves.

According to the women in our study, household work was a reason for health issues and an obstacle to promoting one’s own health, since it leaves the women with too little time to prioritise themselves. Migrant women have previously described a lack of time to promote their own health, due to household work [46]. In this study, the women reasoned that they have a responsibility to change their behaviour to make room for spare time, also expressing that they were inspired by each other when hearing about successful health changes. In addition, when reflecting on possible actions, a suggestion to help each other within the community was brought up. Social support has been used as a strategy among migrant women to stay balanced in stressful situations [28]. Among migrants, social participation can protect against poor mental health when exposed to discrimination, although over 80% of women migrants participate to a low extent socially [47]. This study provided an opportunity to develop social support. The group also included a health promoter that can be regarded as a peer supporter. Community peer support can have a positive effect on health literacy, if following recommendations, such as the health promoter/s being similar to the target group and making the target group participate in social networks [48]. A previous study has described the role of a lay health worker from a migrant population as a participatory researcher and this person’s involvement in research was said to empower the community [49]. Also, Diaz et al*.* recommend that participants are involved in the research process for health interventions intended for migrants [50]. Thus, the migrant women’s actions to support each other and to start the health circle are in line with previous research to promote health.

The women perceived that they did not get proper help from the healthcare and were not listened to, which is why they expressed a will to learn to protest and address politicians. The language barrier can be a reason for migrants not seeking healthcare as much as the general population [51]. Nonetheless, in this group, Arabic-speaking doctors could be available, and some women knew a little Swedish. Further, access to healthcare can be dissimilar for different populations due to racial prejudices but also due to healthcare planning based on the majority of the population [52]. For example, activities for physical activity on prescription were developed for the general population and may not be compatible with a minority population such as migrant women [6]. The construction of gender is acknowledged in most cultures [53] and stereotypical characteristics can cause gender biases [54]. This situation might be troublesome to healthcare personnel to navigate, and gender differences in treatment have occurred [54]. Further, life opportunities might vary between groups due to, for example, gender, class, ethnicity or age [53], and these potentially subordinated social categories might intersect [55]. Consequently, women of colour might be marginalised due to both gender and ethnicity [55], which is why a definition and an analysis of the power relations of social categories are important [56]. Of course this needs to be done with caution in order not to generalise groups within social categories as oppressed [57]. Thus, the mechanisms of the social categories can be important when finding various tools for health promotion adapted to diverse populations [58].

It was apparent that the women who participated in this study already had critical consciousness, but also that they lacked resources to express opposition. Learning to complain about healthcare and making their voices heard, might enable migrant women to impact policy changes. Thus, the dialogues served as a forum to start a process of empowerment and defiance to oppression. This is in line with previous literature, in that health-promotive activities for migrants should focus on supporting them in becoming part of, and being active residents within, the new society [11].

The women identified factors related to health issues that can be considered to be outside of their control, such as the migration process or the imposed sole responsibility for the household. They also felt that they lacked control, being unable to affect their situation, when not getting support from the healthcare as expected. According to Wallerstein and Bernstein, low control leads to ill health, while increased control will lead to better health [22]. Nonetheless, the women considered solutions implying that they are moving towards empowerment. They want to learn about the health system, they want to support each other, and they want to learn how to complain when their healthcare needs are unmet, and they do this through dialogue and the health circle. Empowerment has been described by Wallerstein and Bernstein as: *‘a social action process that promotes participation of people, organizations, and communities in gaining control over their lives in their community and larger society’* [22]. The provision of a forum for dialogue was essential, as well as the role of the health promoter, in creating social inclusion for the process of health promotion. Nikkhah and Redzuan claim that a bottom-up approach to community development will lead to better community empowerment compared to a top-down approach, while a medium level of empowerment could be achieved through the approach of partnership [59]. Of these three approaches, the partnership can best describe the relationship between the community and the other actors in the programme that this study is part of. The participants were the ones to set the agenda in the Future workshops in 2016, where health issues among migrant women were identified. The idea of the health circle came from the researchers, however, and aimed at continuing the dialogue around the issues raised. Knowledge among the participants regarding resources for actions may not have been clear at the time. Thus, the power in a partnership approach may vary during the time of development [59]. Another strategy could have included a second dialogue to explore ideas on actions. Such a strategy might have increased participation and empowerment even more.

### Limitations

The study sample was in the first phase of the programme determined by the community interviews. There is a risk that this method of sampling doesn’t generate a representative sample, although this is a method suitable in hard-to-reach populations [38] and was therefore chosen to be used in this study.

To strengthen generalisability, in the sense of usefulness in other contexts, it is recommended to include more than one story in a story dialogue [33]. However, in this study only one story per group was included, due to time limitation among the participants. The dialogue therefore emerged from that one story, something which might have impacted the richness of the dialogue. Nevertheless, the use of one story led to several important reflections. In this study, we can only conclude that the experiences reported are contextual, with a narrow selection, and have been conducted in a limited community. Still, since there are not very many studies in this group of women, the results may contribute an important perspective, that could be transferred to other groups of migrant women.

The participants and the health promoter were only involved in the first phase of the second-level synthesis, which means that the participatory approach was not possible when merging the three dialogues together, something which could be a limitation. However, different levels of participation are possible in the participatory research process and a lower level of participation should not be an argument for not conducting the research. One could also argue that the first part of the analysis, in which the participants did participate, was the frame for the rest of the analysis.

## Conclusion

Several conditions for health promotion were identified by the participating migrant women through the story dialogues. With the help of the CBPR approach and the health promoters, the participants were involved in the research process from the start and could reflect on what resources they have to promote their own health. The knowledge transfer and learning among the group of women made them want to find strategies to cope with their situation and empowered them as a group to want to learn more about how to change the healthcare system by complaining and acting. The use of the CBPR approach and the story-dialogue method can, together with participation and dialogue, function as an essential foundation for the empowerment process. The health circle can possibly provide a forum for working with these matters and constitute a tool to support migrant health, following recommendations by previous studies.

## Data Availability

The data generated and/or analysed during the current study are not publicly available due to GDPR and secrecy but are available from the corresponding author on reasonable request.

## Abbreviations

WHO: World Health Organization
CBPR: Community-based participatory research
SMS: Short message service
